# Katherine Hohmann

**Published:** 1876-05

**Authors:** Edw. Warren Sawyer

**Affiliations:** Lecturer on Obstetrics, Rush Medical College, Chicago; No. 116 Vincennes Avenue


### KATHERINE HOHMANN.

### A DESCRIPTION OF THIS REPUTED HERMAPHRODITE.

By EDW. WARREN SAWYER, M.D.,

Lecturer on Obstetrics, Rush Medical College, Chicago.

This person, who has been exhibited before the Chicago Society of Physicians and Surgeons, by Prof. Byford; and by myself made the subject of a recent lecture at the Rush Medical College, has for many years been known as an instance of bisexual hermaphrodism. He has also been exhibited before many distinguished medical gentlemen of Europe, and been frequently described in medical literature.*

It will be proper to speak of this creature as being of the masculine gender; inasmuch as he is now a husband, and comports himself in all respects as a male; and because a careful external examination, and interrogation, fail to elicit a single attribute belonging strictly to the feminine sexuality.

He is now fifty-two years of age, was born in Germany,

* Hermaphrodism, From a Medico-Legal Point of View. By Basile Poppesco. Translated from the French by Edw. Warren Sawyer, M.D. W. B. Keen, Cooke & Co. Chicago, 1875.

and christened Katherina. This name he bore till two years since, when, by a decree of the German court, it was changed to Charles. He was reared as a girl; and, as he now asserts, more than once copulated as a female. Menstruation began before the age of twenty, and recurred with measurable regularity. Nothing unusual was noticed in his sexual conformation till the age of thirty-six, when, on account of a strangulated inguinal hernia, he says, he was seen by Simon, of Heidelberg, who operated. Attention was then drawn to his sexual mal-formation; and it was decided that he belonged to the male sex, rather than to the female. From that time he has worn the habiliments of the male. At the age of forty-four, menstruation ceased. The organs which mark him as a male now took on a rapid growth, and soon became a third larger than before. About two years since, he married his present wife, now twenty-six years old; who was advised to escape the dangers of child-bearing, on account of a deformed pelvis. His marital privileges are now so well assumed as to call forth from his wife the highest encomiums. His predilections have always been for the woman. The foregoing is substantially his history, which has been elicited from himself and his companion.

His statue is that of a medium-sized man. The feet are rather small; but the hands are large. The extremities on the right side have a circumference a trifle larger than the left extremities. His right eye is noticeably larger than the left, and of a different shade; while the left pupil is larger than the right. The skin is decidedly less fine than that of the female, and it seems even coarser than that of the cleanly male. His dark hair is very thick, and reaches nearly to his shoulders. His figure is rather a compromise of the male and female; though it has been remarked that the ilia were rather projected outward. The loins and waist are decidedly masculine. His carriage is rather feminine; perhaps on account of his being attired so long as a female. The entire surface is well supplied with hair follicles; the pubic hair extends upward along the median line as far as the umbilicus, as it usually does in the male; and is not abruptly limited, just above the symphysis pubis, as in the female. Moreover, the chin and upper lip are thickly studded with beard, and the sides of the face sparsely so, which is regularly shaven. His axillæ are supplied with hair, and the anus is also surrounded with hair. He has two, well-developed, rather pendulous mammary protuberances; these are surmounted by large, projecting nipples, resting on small, dark, reddish-brown areolæ; the latter are studded with hair.

Concerning the genitalia: On the right side there is a small scrotum, corrugated and covered with hair; in all respects this pouch is quite like the right half of the male scrotum; it encroaches somewhat upon the left side. In the scrotum a good-sized testicle, with its epididymis and vas deferens, can be easily established. On the left side there is no projecting fold suggestive of the scrotum or labium; the surface is here covered with hair. In the normal situation there is a penis which, in the lax condition, measured along the dorsum, is about three inches in length, and of average volume. The penis terminates in a well-formed glans, which is covered superiorly by a lax cutaneous fold like a hood. The virile organ is said to assume a length of six inches during sexual excitement; it does not project outward; because, on account of the absence of the anterior half of the corpora spongiosa, the organ seems to be bound down inferiorly. The bulbous portion of the spongy bodies can be easily made out beneath the penis. Along the dorsum of the penis, there are two, slightly projecting cutaneous folds, with an indurated edge which, in the lax condition, is thrown into many plicæ. These folds converge, and nearly meet at the root of the penis; at this point the skin is so lax that it can be easily lifted from the penis; the folds then strongly resemble the nymphæ of the female. The right nympha is as large as the left. There is a hypospadic slit beneath the glans penis, about a half inch in length. If a sound is placed in this slit, and directed downward, it enters a round opening having the size and appearance of the female meatus urinarius. This opening looks upward, and is on a plane anterior to the root of the penis ; it is brought to within an inch of the end of the organ by the turning downward of the penis. It is through this opening that the catamenial blood is said to have escaped, and through which he copulated as a female ; and which now gives exit to the urine and the liquor seminis. The sound introduced into this meatus can be passed downward about an inch (the subject being in the sitting posture), when it is arrested, and seems separated from the finger externally by a thin septum. If the handle of the sound is now depressed, the instrument passes backward, and slightly upward, some two inches ; it does not enter the bladder, for no urine flows through a catheter thus introduced. The subject does not permit any farther examination with the sound.

In the left groin there is an oval-shaped body, as large as a pullet's egg. This body is movable ; and when seized between the fingers is painful, giving him a sensation which he likens to that which he experiences when his testicle is squeezed. I failed to find any evidence that he had ever submitted to a cutting operation for the relief of a strangulated hernia.

Relying upon the foregoing, which is all that can be elicited from an ordinary examination, the question may well be asked : Is this, indeed, an instance of androgynia, (the association of the essential male and female organs in the same creature,) and not a mal-formed man, with cryptorchidia and hypospadias? It seems to me, that, from this examination alone, one is forced to decide upon a male sexuality. Even the presence of the pendulous mammæ does not preclude this ; for instances are known of men who have had mammæ sufficiently developed to afford nourishment to a child.* Especially has

* Vide Flint's Physiology of Man, Vol. 3, page 73. D. Appleton & Co. New York, 1870.

this unusual mammary development been noticed if the male is the subject of cryptorchidia, or has been emasculated in early youth.

The supposed female sexuality of this creature is founded solely upon the assumed menstruation. There can be no doubt but that he is thought to have menstruated ; for in the scientific description of him in the *Lo Sperimentale*, referred to by Poppesco,* the function of menstruation is assumed to have existed. Moreover, men as eminent as Recklinghausen, Kölliker and Scanzoni, are reported to have said that they have seen blood escape from this man's urethra ; and that this blood possessed all the microscopical appearances of catamenial blood.

A doubt has been raised by some as to the possibility of copulation being accomplished through this narrow meatus. The fact that there are authenticated instances of the female urethra being the habitual avenue of copulation, should protect this man's assertion from total discredit.

But in view of the facts : first, that this creature does possess the essential characteristics of the male—for his semen, which has been examined by Virchow, contains numerous spermatozoids ; second, that there is a possibility that the blood which escaped periodically from the urethra may not have been true menstrual blood—for this creature soon realized that he was the subject of great wonder, and might have easily inflicted some slight wound of the canal, sufficient to have produced an oozing of blood ; moreover, the microscropical appearances of menstrual blood are, by no means, thought by all to be distinctive. In view of these facts, I say, it seems more rational to call this a mal-formed man, with hypospadias and incomplete cryptorchidia. Or, at most, we must allow this creature to remain unclassed, until a *post-mortem* examination shall establish, beyond doubt, the presence of the essential organs of the female, the uterus or the ovary.

* Poppesco, op. cit., page 40.

No. 116 Vincennes Avenue.

